# Immunorecovered but Exhausted: Persistent PD-1/PD-L1 Expression Despite Virologic Suppression and CD4 Recovery in PLWH

**DOI:** 10.3390/biomedicines13081885

**Published:** 2025-08-03

**Authors:** Bogusz Aksak-Wąs, Karolina Skonieczna-Żydecka, Miłosz Parczewski, Rafał Hrynkiewicz, Filip Lewandowski, Karol Serwin, Kaja Mielczak, Adam Majchrzak, Mateusz Bruss, Paulina Niedźwiedzka-Rystwej

**Affiliations:** 1 Department of Infectious, Tropical Diseases and Acquired Immunodeficiency, Pomeranian Medical University in Szczecin, 70-204 Szczecin, Poland; 2 Department of Biochemical Science, Pomeranian Medical University in Szczecin, 70-204 Szczecin, Poland; 3 Institute of Biology, University of Szczecin, 70-453 Szczecin, Poland; 4 Center for Experimental Immunology and Immunobiology in Infectious Diseases and Cancer, University of Szczecin, 70-453 Szczecin, Poland; 5 Regional Center for Digital Medicine, Department of Genomics and Forensic Genetics, Faculty of Medicine, Pomeranian Medical University in Szczecin, 70-204 Szczecin, Poland

**Keywords:** HIV, PD-1, immune reconstitution, immune exhaustion CD4, CD8

## Abstract

**Background/Objectives:** While ART effectively suppresses HIV viremia, many PLWH exhibit persistent immune dysfunction. This study aimed to assess immune recovery and immune exhaustion (PD-1/PD-L1 expression) in newly diagnosed versus long-term ART-treated individuals. **Methods:** We analyzed 79 PLWH: 52 newly diagnosed individuals (12-month follow-up) and 27 long-term-treated patients (Ukrainian refugees). Flow cytometry was used to evaluate CD4+ and CD8+ counts, the CD4+/CD8+ ratio, and PD-1/PD-L1 expression on CD3+, CD4+, and CD19+ lymphocytes. ART regimen and HIV subtype were included as covariates in linear regression models. **Results:** At 12 months, CD4+ counts were similar between groups (median 596.5 vs. 621 cells/μL, *p* = 0.22), but newly diagnosed patients had higher CD8+ counts (872 vs. 620 cells/μL, *p* = 0.028) and a lower CD4+/CD8+ ratio (0.57 vs. 1.05, *p* = 0.0027). Immune exhaustion markers were significantly elevated in newly diagnosed individuals: CD4+ PD-1+ T cells (24.4% vs. 3.85%, *p* = 0.0002) and CD3+ PD-1+ T cells (27.3% vs. 12.35%, *p* < 0.0001). Linear regression confirmed group membership independently predicted higher CD3+ (β = +21.92, *p* < 0.001), CD4+ (β = +28.87, *p* < 0.0001), and CD19+ (β = +8.73, *p* = 0.002) percentages. Lipid parameters and SCORE2 did not differ significantly. **Conclusions:** Despite virologic suppression and CD4+ recovery, immune exhaustion markers remain elevated in newly diagnosed PLWH, suggesting incomplete immune normalization. Traditional parameters (CD4+ count and CD4+/CD8+ ratio) may not fully capture immune status, warranting broader immunologic profiling in HIV care.

## 1. Introduction

Antiretroviral therapy (ART) has transformed HIV disease into a manageable chronic condition by suppressing viral replication and enabling recovery of immune function [1]. Virologic control with ART typically leads to increased CD4+ T-cell counts and improvement of immune competence, which in turn dramatically reduces the risk of AIDS-related opportunistic illnesses and mortality [2,3]. This restoration of immune function—often termed immune recovery—is a central clinical goal in HIV management, as robust CD4+ T-cell recovery underlies the long-term health and survival of people living with HIV (PLWH). Immune reconstitution is usually evaluated by immunological indices, principally the CD4+ T-lymphocyte count. Patients who achieve a CD4+ count above key thresholds (e.g., surpassing 200 cells/μL is associated with the highest risk for opportunistic infections, and ideally >500 cells/μL indicates approaching the lower limit of normal) are considered to have a favorable immune recovery [4,5]. In recent years, the CD4+/CD8+ ratio has also emerged as an important marker of immune reconstitution [6,7]. In people with untreated HIV, this ratio is typically inverted (well below 1.0), and its gradual normalization on ART reflects a return to immune balance. PLWH who fail to restore a normal CD4+/CD8+ ratio despite adequate CD4+ count gains often exhibit persistent immune dysfunction and inflammation [5]. Notably, a persistently low CD4+/CD8+ ratio (e.g., <0.4–0.5) during ART is associated with an elevated risk of non-AIDS morbidity and mortality, independent of absolute CD4+ counts [5]. Even with sustained virologic suppression, an estimated 10–40% of individuals experience incomplete CD4+ T-cell recovery and are classified as immunologic non-responders (INRs) [2,8,9,10]. These patients fail to reach expected CD4+ count thresholds or functional recovery and remain at heightened immunological risk. Multiple factors contribute to such variability in immune restoration, such as HIV subtype, time of HIV infection, and genetic background [11]. Immune restoration as well as other factors may influence mortality ratio as well [12,13,14]. Low pretreatment CD4+ nadir is a well-recognized predictor of blunted recovery, as starting ART at advanced immunosuppression limits the capacity for full rebound [2,15]. Older age is another key factor: older PLWH tend to have slower CD4+ T-cell gains and persistently lower CD4+/CD8+ ratios than younger individuals, correlating with higher mortality and more comorbid conditions [11]. Co-infections can further hinder immune reconstitution—for example, concomitant active tuberculosis or hepatitis B/C infection has been linked to higher rates of immunologic failure, likely by driving chronic immune activation and tissue damage [16]. On a mechanistic level, persistent inflammation and immune activation despite viral suppression are thought to be major impediments to CD4+ recovery [8]. Immune reconstitution may be constrained by factors such as thymic dysfunction (limiting new T-cell output), latent HIV reservoirs and low-level viral replication, disruption of gut mucosal integrity with microbial translocation, and other sources of ongoing immune system stimulation that lead to continued CD4+ T-cell turnover [8,17,18]. Emerging evidence also implicates dysregulation of innate immunity in suboptimal immune recovery. For instance, recent studies highlight that aberrant natural killer cell interactions and receptor-ligand profiles in INRs can impair CD4+ T-cell expansion, underscoring the multifaceted immunological crosstalk involved in reconstitution [19]. This broad array of host and viral factors underpins the complex heterogeneity observed in immune recovery outcomes.

The clinical consequences of suboptimal immune reconstitution are considerable. INRs who remain with low CD4+ counts are at continued risk for AIDS-defining opportunistic infections and other immunodeficiency-related complications, even if HIV viremia is controlled [8]. Moreover, individuals with low CD4+/CD8+ ratios have shown increased rates of cardiovascular and renal events, and immune non-responders in general exhibit more rapid progression of age-related comorbidities than those who fully reconstitute [20,21,22]. In epidemiologic studies, a poor immune reconstitution profile (e.g., CD4+ count remaining <350–500/μL or CD4+/CD8+ ratio < 0.5) correlates with higher all-cause mortality and serious non-AIDS events, reflecting the lasting impact of immune deficiency and inflammation on overall health [5,8]. Thus, failure to normalize immune parameters under ART can lead to “immune aging” and excess morbidity, even as the virus itself remains suppressed.

### 1.1. The PD-1/PD-L1 Pathway in HIV

Research has demonstrated that PD-1 expression is markedly increased in individuals infected with HIV, leading to compromised T-cell activity and facilitating immune system evasion [23]. Studies have also shown that PD-1 mediates HIV immune evasion through the MAPK/NF-κB pathways [24]. Moreover, it was demonstrated that the PD-1 monoclonal antibody (pembrolizumab) can significantly upregulate HIV-1 LTR transcription, suggesting that PD-1 not only contributes to immune escape during HIV infection but may also promote the persistence of latent infection [24]. Other studies have indicated that immune checkpoint blockade therapies targeting PD-1 and/or CTLA-4 are unlikely to achieve HIV remission in individuals with sustained viremia unless combined with supplementary therapeutic strategies [25]. What is also known is that PD-1 blockade restores T-cell function and activates latent HIV, suggesting potential therapeutic strategies for HIV treatment. As a result, exploring the role of PD-1 in the context of HIV infection and elucidating the mechanisms driving its expression are essential steps toward enhancing immune function in HIV-positive individuals [23].

### 1.2. Study Hypothesis

We hypothesize that a more comprehensive understanding of the determinants and kinetics of immune reconstitution will enable better prediction of which patients are at risk for suboptimal recovery and inform targeted strategies to improve immune outcomes. In this context, studying immune reconstitution as a dynamic, multifactorial phenomenon—integrating clinical, virologic, and immunologic parameters—is crucial to ultimately devise interventions that ensure durable and complete immune restoration in all people living with HIV.

Subgroup-specific effects of ART regimens and HIV subtypes on immune reconstitution were investigated separately and are presented in a prior study [1].

## 2. Materials and Methods

Longitudinal data were collected from 79 patients (52 patients in the newly diagnosed group and 27 patients in the long-term treatment group) followed up at the Department of Acquired Immunodeficiency, Pomeranian Medical University in Szczecin, Poland. The study protocol was approved by the Bioethical Committee of the Pomeranian Medical University (approval number KB-006/40/2023; date: 31 May 2023). Written informed consent was obtained from all participants, and the data were fully pseudoanonymized before being used in statistical analyses.

Patients were divided into two observational cohorts. The first consisted of patients newly diagnosed with HIV infection, followed for 12 months, and the second consisted of a group of long-term-treated patients entering care at a random point of infection. The group of people undergoing long-term treatment (i.e., at least 2 years before inclusion in the study) was a group of PLWH refugees from Ukraine. Due to the geopolitical situation in Poland, the sudden influx of patients from abroad who started treatment a long time ago allowed for the selection of a control group for the study.

The long-term treatment group consisted entirely of adult PLWH (≥18 years of age) who were refugees from Ukraine and had entered care at our institution between 2022 and 2023. Inclusion criteria for this group were a confirmed duration of antiretroviral therapy of at least two years, virologic suppression (HIV-RNA < 200 copies/mL) at entry, and no documented interruptions in treatment. Detailed prior clinical data were variably available due to displacement-related healthcare disruption, precluding further stratification by ART duration. Detailed records of exact time since HIV diagnosis, nadir CD4+ counts, or ART history were variably available. No pediatric or adolescent individuals were included.

The entry criteria were all patients with newly diagnosed HIV infection attending the local clinic, regardless of gender, age, HIV viral load, or CD4+ lymphocyte count. There were no criteria for exclusion from the analyses. Patients were assessed after 12 months (+/−3 months) by assessing variables such as the CD4+/CD8+ immunoprofile and the percentage of PD-1 and PD-1 ligand receptors on CD3+CD4+, CD3+CD8+, and CD19+ lymphocytes; they were then compared with the baseline data of the group of “long-term-treated patients”. HIV subtype was assessed at entry into care for newly diagnosed individuals and from proviral DNA for the long-term treatment group.

For analyses of immune exhaustion, the criterion of the effective antiretroviral treatment was set. The patient had to achieve the criterion of treatment success within 6 months, defined as an HIV RNA viral load < 200 copies/mL, and maintain undetectable viremia throughout the observation period. Patients in the long-term treatment group were required to have a viral load of <200 copies/mL at the time of baseline analysis.

### 2.1. Immunophenotyping

**Figure 1:**
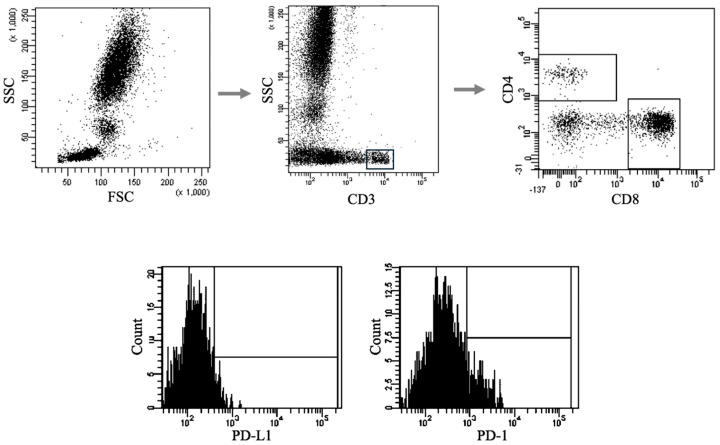
Sample analysis of immunophenotyping for PD-1/PD-L1 expression on T and B lymphocytes introducing the gating strategy. Representative flow cytometry plots showing the gating strategy used to identify lymphocyte populations and assess expression of immune checkpoint markers. Top row (from left): initial gating of lymphocytes based on forward scatter (FSC) and side scatter (SSC) parameters → selection of CD3+ T cells → further discrimination of CD4+ and CD8+ T-cell subsets. Bottom row: histograms illustrating representative fluorescence intensity distributions of PD-L1 and PD-1 expression on gated lymphocyte subsets.

Peripheral blood samples for immunophenotyping were collected in tubes containing ethylenediaminetetraacetic acid (EDTA). To identify T and B lymphocytes expressing PD-1/PD-L1, cells were analyzed using unstained controls, fluorescence-minus-one (FMO) controls, and samples stained with monoclonal antibodies conjugated to fluorescent dyes (Becton Dickinson, East Rutherford, NJ, USA). Each sample was incubated with 20 μL of the respective monoclonal antibody for 20 min at room temperature, followed by 30 min at 4 °C in the dark. Post-staining, cells were treated with a lysing solution (Becton Dickinson) and incubated for 15 min at 4 °C in the dark, then washed twice with phosphate-buffered saline. Lymphocyte subsets were identified using standard cell labeling and gating strategies, which included doublet exclusion (FSC-A vs. FSC-H) and dot-plot analysis (FSC vs. SSC). Gating strategies are illustrated in Figure 1. Data acquisition was performed using an eight-color FACSCanto II flow cytometer (BD Biosciences, Franklin Lakes, NJ, USA), and data were analyzed with Diva v 9.0.1 software (BD Biosciences). For each sample, a minimum of 10,000 events were recorded, with up to 50,000 acquired when available.

### 2.2. Statistical Analysis

To compare continuous variables between two clinical groups, the non-parametric Mann–Whitney U test was employed. This test was selected due to its suitability for data that do not meet the assumption of normal distribution and for small and unequal sample sizes. The analyzed variables included selected biochemical parameters (e.g., CD4/CD8 ratio, LDL, TAG, and total cholesterol) and immunological markers (e.g., Th (CD4+), T (CD3+), and B (CD19+) cells and their PD-1/PD-L1 expression). Comparisons were conducted independently for each variable. Results were presented as medians and interquartile ranges (IQR). For each continuous variable, linear regression analysis was performed with clinical group membership as the primary independent variable. Each model included three covariates: type of antiretroviral therapy (ART category) and HIV subtype. The aim was to estimate the independent effect of group affiliation on selected biochemical and immunological parameters. For each model, the coefficient of determination (R^2^ and adjusted R^2^), F-statistic, and *p*-values for individual predictors were calculated. Statistical effects were considered significant at *p* < 0.05. For variables showing significant differences, boxplots were generated to illustrate the distribution of results across groups. All analyses were conducted using Python v 3.11 with the statsmodels, pandas, matplotlib, and seaborn libraries.

## 3. Results

### 3.1. Patient Characteristics

#### 3.1.1. Newly Diagnosed Group

The study group consisted of 52 persons with male predominance (n = 34; 65.4%), with an average age of 39.0 (IQR: 32–46) years. Subtype A6 was the most prevalent with n = 25 (55.6% of patients), followed by subtype B with n = 18 (40.0%) and subtypes G and C with n = 1 (2.2%) each (subtype was known in 45 individuals).

For ART, a predominantly INSTI + TAF-based regimen was used (n = 30, 57.7%), followed by INSTI + TDF (n = 14, 26.9%) and other (NNRTI or PI-based regimen + TDF or TAF) (n = 6 (11.5%)).

#### 3.1.2. Long-Term Treatment Group (Ukraine Refugees Group)

The study group consisted of 27 persons with male predominance (n = 14; 51.9%), with an average age of 42.0 (IQR: 35–48) years. Subtype was known in 18 individuals, with A6 being most prevalent with n = 14 (77.8% of patients), followed by subtype B with n = 3 (16.7%) and subtype F1 with n = 1 (5.6%).

For ART, a predominantly INSTI + TDF-based regimen was used (n = 23, 88.5%), followed by other (NNRTI or PI-based regimen; n = 3 (11.5%)). There was no statistically significant difference in age between groups (42 vs. 39 years, *p* = 0.19).

### 3.2. Biochemical and Immunological Data

For the clinical assessment of both groups, the checkpoint at 12 months of observation was assessed as the time needed for the HIV-related inflammatory response to subside for newly diagnosed patients and as the point of entry into care in Poland for the long-term treatment group. Clinical data did not differ significantly for both groups at the given time points as outlined in Table 1. However, there was a statistical tendency noted for higher TC concentration in newly diagnosed patients.

### 3.3. Assessment of the Immune Response in Both Subgroups

When analyzing the immune reconstitution in both groups, no differences were found in the number of CD4+ lymphocytes, but a statistically significant difference was observed with a higher number of CD8+ lymphocytes in the group of newly diagnosed patients (assessment at 12 months). Similarly, it was shown that the group of refugees from Ukraine (long-term-treated patients) had a statistically higher CD4+/CD8+ ratio, which may indicate that 12 months of observation was too short to achieve full immune reconstruction, despite the lack of differences in the number of CD4+ lymphocytes in both of these subgroups. The exact numerical data of immune reconstruction are shown in Table 2.

### 3.4. Assessment of Immune Exhaustion in Both Subgroups

In the assessment of immune exhaustion, markers PD-1 and PD-1 ligand on B lymphocytes (CD19+), all T lymphocytes (CD3+), and T helper (CD3+ CD4+) were assessed. Significantly higher values were demonstrated in all of these analyses for the 12-month follow-up point in newly diagnosed patients compared to the long-term treatment group.

The greatest differences were observed in the immune exhaustion receptors on Th_CD4+ lymphocytes, with PD-1 found in 24.4% (IQR: 18.95–32.70) in the newly diagnosed group in comparison to 3.85% (IQR: 0.00–21.75) in long-term-treated individuals (*p* = 0.0002).

All comparative data of immune exhaustion receptors are presented in Table 3.

### 3.5. Linear Regression Evaluation

To estimate the independent effect of the group on the level of individual biochemical and immunological parameters, linear regression analysis was performed for each of the dependent variables, in which the main variable was the membership in a specific clinical group. Two control variables were included in the models: type of antiretroviral therapy used (ART category) and HIV subtype. Results are given in Table 4.

Although the models for LDL, TAG, total cholesterol, non-HDL cholesterol, and systolic blood pressure were well fitted, the effect of the clinical group did not reach statistical significance (*p* > 0.05). Thus, none of the analyzed lipid-related parameters showed a statistically significant independent association with clinical group membership.

Immunological indices related to helper T lymphocytes (Th_CD4+), total T cells (T_CD3+), and B_CD19+ cells were analyzed. Newly diagnosed individuals exhibited a significantly lower CD4+/CD8+ ratio (β = −0.44, *p* = 0.014), suggesting impaired immune reconstitution. At the same time, a significantly higher proportion of Th_CD4+ cells (β = +28.87, *p* < 0.001), total T_CD3+ cells (β = +21.92, *p* < 0.001), and B_CD19+ cells (β = +8.73, *p* = 0.00154) was observed in this group. The percentage of Th_CD4+ cells expressing PD-L1 showed a near-significant increase (β = +2.29, *p* = 0.069), possibly indicating heightened immune activation, while B_CD19+ PD-1 expression showed a non-significant negative trend (β = −2.10, *p* = 0.097). No significant differences were found for T (CD3+) PD-1, Th (CD4+) PD-1, or B (CD19+) PD-L1 expression (*p* > 0.1). These findings suggest that newly diagnosed patients exhibit a more activated immune profile, as evidenced by increased Th and B lymphocyte levels, independently of ART regimen and HIV subtype.

## 4. Discussion

### 4.1. Summary of Findings

In this study, we compared markers of immune reconstitution and exhaustion between two groups of people living with HIV (PLWH): 52 individuals newly diagnosed with HIV (followed for 12 months after initiating antiretroviral therapy, ART) and 27 individuals on long-term suppressive ART. We assessed T-lymphocyte counts (CD4+ and CD8+), the CD4+/CD8+ ratio, and expression of the immune checkpoint molecules PD-1 and PD-1-L on major lymphocyte subsets (T_CD3+, Th_CD4+, and B_CD19+ cells). After 12 months of ART, the newly treated patients showed significant immune reconstitution; the median of Th_CD4+ rose above 500 cells/μL, resulting in partial reconstruction of the CD4/CD8 ratio as well. However, compared to the long-term treatment group, the newly diagnosed group showed much higher percentages of immune exhaustion receptors on all analyzed cells at the 12-month checkpoint. Interestingly, this did not translate into an increase in SCORE2 cardiovascular risk [26]. In the analysis controlling for two covariates—ART category and HIV subtype—clinical group emerged as a significant predictor of immunological parameters, particularly the proportions of Th (CD4+), T (CD3+), and B (CD19+) lymphocytes.

The statistically significant reduction in the CD4+/CD8+ ratio (β = −0.44, *p* = 0.014) observed in the newly diagnosed group, despite similar CD4+ T-cell counts between groups, reflects an incomplete restoration of immune balance within the first year of ART. This inversion suggests persistent CD8+ T-cell expansion or delayed contraction of effector populations, which has been associated with residual immune activation and a higher risk of non-AIDS comorbidities [5]. Moreover, the significantly elevated percentages of CD3+ (β = +21.92, *p* < 0.001), CD4+ (β = +28.87, *p* < 0.001), and CD19+ (β = +8.73, *p* = 0.002) lymphocytes in the newly diagnosed group may indicate robust cellular repopulation following ART initiation, potentially driven by early homeostatic proliferation and immune system rebound. However, this increase should be interpreted with caution, as it may not necessarily equate to functional normalization and could also reflect a reactive, activated immune phenotype. These differences persisted despite adjustment for HIV subtype and ART regimen, supporting a distinct immunologic profile during early treatment phases. Thus, while the overall increase in lymphocyte subsets suggests immune activation and expansion, the failure to normalize the CD4+/CD8+ ratio points to an ongoing imbalance that may predispose patients to chronic inflammation or immune dysregulation.

### 4.2. Interpretation of Immune Exhaustion During ART Therapy in PLWH

Our findings highlight that immune checkpoint markers of T-cell exhaustion remain abnormally elevated in PLWH despite 12 months of virologically suppressive ART [1]. PD-1 (programmed cell death-1) and its ligand PD-L1 are central mediators of T-cell exhaustion in chronic viral infections [27]. Persistent antigen exposure (from HIV itself or other co-pathogens) upregulates PD-1 on T cells, delivering inhibitory signals that curb T-cell proliferation and cytokine production [9,27]. The result is a functionally “exhausted” T-cell population with reduced ability to clear virus. In our study, the sustained high PD-1 expression on CTLs_CD8+ cells during ART is a clear indicator of such exhaustion, even in the context of controlled plasma viremia. This is consistent with prior observations that high percentages of PD-1 receptors on CTLs_CD8+ cells remain similar to pre-therapy levels after 1–2 years of ART [28]. In contrast, PD-1 levels on Th_CD4+ cells showed an initial drop after ART initiation (likely reflecting relief from high viremia-driven activation) but then plateaued, remaining above normal ranges [28]. The persistence of a high percentage of PD-1 T cells despite viral suppression suggests ongoing low-level antigenic stimulation or a durable imprint of exhaustion. Indeed, even with early initiation of ART, complete reversal of T-cell exhaustion phenotypes may not occur [27]. A recent immunologic analysis noted that suppressive ART does not fully normalize expression of inhibitory receptors like PD-1, TIM-3, and TIGIT—even in individuals treated during acute HIV infection [27,29]. Our data support this: after one year of ART, patients still exhibited heightened PD-1/PD-L1 levels relative to expected healthy values. From a mechanistic standpoint, this enduring PD-1 elevation has important implications. Lymphocyte T cells with a high percentage of PD-1 receptors in treated HIV infection tend to have an impaired capacity to proliferate or secrete IL-2/IFN-γ [28]. They also harbor more HIV DNA, linking exhaustion to the residual viral reservoir [28]. Notably, the expression of PD-1 on T cells is not merely a bystander epiphenomenon but appears to carry prognostic weight. Previous studies have shown that among ART-suppressed patients, those with higher PD-1 expression have a larger intact proviral reservoir and are more prone to viral rebound if therapy is interrupted [27]. Chronic HIV infection is known to drive aberrant B-cell activation and an “exhausted” memory B-cell phenotype [30]. These exhausted B cells often overexpress inhibitory receptors (including PD-1 and PD-L1) and show functional deficits in antibody production. Prior research has documented that HIV-infected individuals have an expanded population of PD-L1–expressing B cells compared to HIV-negative controls [31]. In summary, the immunophenotypic data suggest that while ART achieves “immune restoration” in terms of quantity (Th_CD4+ count recovery), it does not wholly restore immune quality. Key pathways of T-cell and B-cell exhaustion remain active. This ongoing immune exhaustion, as evidenced by PD-1/PD-L1 upregulation, likely reflects both the intrinsic memory imprinting from chronic HIV antigen exposure and extrinsic drivers like co-infections (e.g., CMV) and inflammatory microbial products that persist in treated HIV. The clinical consequence is an immune system that, even under full viral suppression, retains features of chronic activation and functional impairment.

While our findings clearly demonstrate elevated expression of immune checkpoint markers (PD-1 and PD-L1) in newly diagnosed individuals after 12 months of ART, we acknowledge that equating this with “immune exhaustion” may be an oversimplification. Immune exhaustion is a multifaceted functional state characterized not only by upregulation of inhibitory receptors but also by impaired proliferative capacity, diminished cytokine production (e.g., IFN-γ, IL-2), and altered transcriptional and epigenetic profiles [27,28]. Our study utilized surface expression of PD-1 and PD-L1 as immunophenotypic correlates of exhaustion based on robust evidence that sustained expression of these markers is associated with T-cell dysfunction and poorer HIV-specific immune responses—even under long-term ART suppression [23,28]. However, we did not assess cellular functionality through antigen-specific stimulation assays or intracellular cytokine measurements, which are necessary for confirming true functional exhaustion. Despite this limitation, our findings provide meaningful insights into the persistent immune dysregulation under ART, as PD-1/PD-L1 expression remains a relevant marker of chronic immune activation and a predictor of reservoir persistence. Future studies integrating phenotypic, transcriptional, and functional analyses are warranted to better characterize the continuum between activation, dysfunction, and exhaustion in PLWH on ART.

### 4.3. Possible Explanations for Findings

Several biological mechanisms may explain why immune exhaustion persists and why long-term-treated PLWH continue to show immunologic disparities compared to those who are newly treated. In our newly diagnosed cohort, although we did not stratify by baseline Th_CD4+, those who started with less advanced disease might be expected to show faster immune normalization. The fact that significant exhaustion remained after 12 months, however, suggests that even prompt treatment cannot immediately reset the immune system’s regulatory circuits. A second explanation is the presence of the HIV reservoir and ongoing antigenic stimulation. Even under suppressive ART, low-level HIV antigen production may continue in tissues. There is evidence that HIV proteins can be sporadically expressed by latently infected cells, especially in lymphoid tissue sanctuaries, without causing systemic viremia. T cells continually encountering these antigens could maintain expression of PD-1 and other checkpoints [32,33].

While univariate analysis (Table 3) demonstrated significantly elevated levels of immune exhaustion markers (PD-1 and PD-L1) on CD3+, CD4+, and CD19+ lymphocytes in newly diagnosed individuals after 12 months of ART, these differences did not remain statistically significant in the multivariate regression models (Table 4) after adjusting for ART regimen and HIV subtype. This divergence underscores the influence of treatment-related and virological factors on immune exhaustion profiles. In particular, the more prevalent use of INSTI + TAF-based regimens and the higher proportion of subtype B infections in the newly diagnosed group could partially account for the observed immune activation and checkpoint marker expression. Recent studies have shown that different ART regimens may exert heterogeneous immunomodulatory effects beyond virologic suppression, influencing both immune activation and exhaustion pathways [2,27,34]. Similarly, HIV subtypes have been associated with distinct patterns of immune recovery and residual immune activation [11]. The attenuation of group differences in the adjusted model suggests that the elevated PD-1/PD-L1 expression in the newly diagnosed group may, at least in part, reflect these confounding variables rather than being solely attributable to the stage of treatment. Nevertheless, the consistently elevated checkpoint expression seen in the unadjusted analysis remains clinically relevant, particularly as the absolute levels of exhaustion markers in newly diagnosed individuals are substantially higher than those typically seen in long-term ART patients, indicating incomplete immunologic normalization. Therefore, while the statistical significance diminishes after covariate adjustment, the biological signal of persistent immune exhaustion remains notable and may have clinical implications in terms of long-term immune competence and reservoir persistence.

### 4.4. Limitations of the Study

Our study has several limitations that should be acknowledged:

The first is the small sample size. With 79 participants, the study may be underpowered to detect subtle associations or to generalize findings to the broader HIV-infected population. Larger studies are needed to validate these observations.

The other limitation is the absence of study matching. Due to the geopolitical situation, it was not possible to match the groups 1:1, and although both groups did not differ significantly in biochemical data, differences in HIV subtype or approach to ARV therapy could have had an impact on the results.

Additionally, the use of Ukrainian refugees as the long-term-treated control group, while practically advantageous due to their distinct treatment duration and availability, may introduce unmeasured confounding variables. These individuals may have experienced chronic stress related to forced migration and geopolitical instability, which can modulate immune function independently of HIV infection or ART status. Moreover, limited access to historical treatment records, potential variations in adherence prior to arrival, and differences in baseline nutritional status may further impact immunological parameters, including PD-1/PD-L1 expression. Although all individuals in this group met strict virologic suppression criteria at the time of enrollment, we recognize that these non-biomedical factors may influence immune exhaustion profiles. Therefore, caution is warranted in interpreting between-group comparisons, and future studies with more homogenous and well-documented cohorts are needed to validate our findings.

Furthermore, the use of an opportunistic control group composed of long-term ART-treated Ukrainian refugees presents an important methodological constraint. No baseline immunologic or clinical data were available for this group, preventing any assessment of intra-individual immune trajectories or direct evaluation of longitudinal ART effects. As such, the study design does not allow for conclusions to be drawn regarding the temporal impact of ART on immune exhaustion or reconstitution parameters within the long-term treatment group. Instead, all between-group comparisons should be interpreted as cross-sectional associations rather than longitudinal effects. While inclusion criteria ensured virologic suppression and sustained ART for at least two years, the absence of paired baseline measurements limits the capacity to infer causality or treatment-attributable changes. Future longitudinal studies incorporating baseline and follow-up immune profiling across all groups are necessary to substantiate these preliminary findings.

Another limitation is the short follow-up period. A 12-month follow-up may not be sufficient to observe long-term patterns of immune exhaustion and PD-1/PD-L1 expression changes. Extended longitudinal studies could provide more comprehensive insights.

While therapeutic modulation of PD-1/PD-L1 has shown potential in oncology and HIV cure research, our study did not involve intervention or treatment targeting this pathway. Therefore, we respectfully do not cite case reports describing pembrolizumab use in non-HIV settings, such as cutaneous reactions in lung cancer patients. Nevertheless, the persistent elevation of PD-1/PD-L1 we observed may identify a subset of PLWH who could benefit from future immune-based therapies. Clinical trials investigating checkpoint blockade in ART-treated individuals are ongoing and may clarify the therapeutic implications of our findings [28].

The findings from our study raise the question of whether immune exhaustion markers should be incorporated into routine monitoring of ART-treated PLWH. While current guidelines emphasize CD4+ count and viral suppression, persistent elevation of PD-1/PD-L1—even in virologically suppressed individuals—may signal residual immune dysfunction and increased risk for comorbidities. Although not yet recommended in clinical practice, further validation of these markers could support their role in identifying individuals who may benefit from adjunctive immunotherapies or closer monitoring for immune aging.

## 5. Conclusions

In conclusion, the comparative analysis of newly diagnosed versus long-term ART-treated PLWH reveals that while antiretroviral therapy robustly reconstitutes CD4+ T-cell counts, it only partially alleviates immune exhaustion. The persistence of immune exhaustion may contribute to the elevated risk of non-AIDS comorbidities (cardiovascular disease, malignancies, etc.) seen in well-treated PLWH. In the future, when thinking about immune reconstruction, a wider range of parameters should be taken into account than just the CD4+ value or the CD4+/CD8+ ratio.

## Figures and Tables

**Table 1:** Comparative biochemical data for long-term-treated patients vs. newly diagnosed individuals.

	Long-Term-Treated Patients (Ukraine Refugees Group) (n = 27)	Newly Diagnosed Indyviduals (n = 52)	*p* Value
Total cholesterol (median, IQR, mg/dL)	172	165	0.6344
	155.50 to 194.25	145.50 to 204.50	
Non-HDL cholesterol (median, IQR, mg/dL)	124.8	116.55	0.9753
	103.33 to 136.55	90.20 to 152.50	
HDL cholesterol (median, IQR, mg/dL)	52.2	49.15	0.1537
	43.80 to 66.28	41.85 to 56.25	
LDL cholesterol (median, IQR, mg/dL)	120	123	0.8443
	106.00 to 134.75	87.50 to 148.50	
Triacylglycerols (median, IQR, mg/dL)	86	96	0.0551
	62.50 to 98.50	76.50 to 157.50	
SCORE 2	0.02	0.02	0.6918
	0.0025 to 0.040	0.00 to 0.040	

**Table 2:** Immune reconstruction data in compared datasets.

	Long-Term-Treated Patients (Ukraine Refugees Group) (n = 27)	Newly Diagnosed Individuals (n = 52)	*p* Value
Th_CD4+ (median, IQR, cells/μL)	621	596.5	0.22
	487 to 882	285.0 to 811.0	
CTLs_CD8+ (median, IQR, cells/μL)	620	872	0.028
	494.0 to 936.00	603.0 to 1250.75	
Th_CD4+/CTLs_CD8+ (median, IQR, ratio)	1.05	0.57	0.0027
	0.69 to 1.55	0.35 to 1.05	

**Table 3:** Immune exhaustion parameters is tested individuals.

	Long-Term-Treated Patients (Ukraine Refugees Group) (n = 20)	Newly Diagnosed Individuals (n = 52)	*p* Value
B_CD19+ PD-1 (median, IQR, %)	0	0.6	0.0418
	0.00 to 4.10	0.30 to 1.20	
B_CD19+ PD-L1 (median, IQR, %)	0	0.2	0.0044
	0.00 to 0.00	0.00 to 0.60	
T_CD3+ PD-1 (median, IQR, %)	12.35	27.3	<0.0001
	3.75 to 18.35	21.10 to 36.40	
T_CD3+ PD-L1 (median, IQR, %)	0	0.8	0.0029
	0.00 to 1.65	0.20 to 5.00	
Th_CD4+ PD-1 (median, IQR, %)	3.85	24.4	0.0002
	0.00 to 21.75	18.95 to 32.70	
Th_CD4+ PD-L1 (median, IQR, %)	0	0.45	<0.0001
	0.00 to 0.00	0.050 to 5.30	

**Table 4:** Linear regression evaluation of given different clinical and immunological data.

Variable	Coefficient (β)	*p*-Value
T_CD3+ (%)	+21.92	<0.001
T_CD3+ PD-1 (%)	+8.03	0.11
T_CD3+ PD-1L (%)	−0.043	0.97
CTLs_CD8+ cells	+167.33	0.27
Th_CD4+ cells	−165.50	0.097
Th_CD4+ (%)	+28.87	<0.0001
Th_CD4+ PD-1 (%)	+5.11	0.42
Th_CD4+ PD-L1 (%)	+2.26	0.069
B_CD19+ (%)	+8.73	0.002
B_CD19+ PD-1 (%)	−2.10	0.097
B_CD19+ PD-L1 (%)	−0.02	0.97
CD4+/CD8+	−0.44	0.014
RR systolic	−4.07	0.33
LDL	+10.53	0.35
Non-HDL	+7.92	0.36
TAG	+26.23	0.18
Total cholesterol	2.57	0.81

## Data Availability

As the publication process for all associated articles is in progress, raw data is available upon request from the authors. For data requests, please contact the corresponding author: karolina.skonieczna.zydecka@pum.edu.pl.
